# Loneliness predicts decreased physical activity in widowed but not married or unmarried individuals

**DOI:** 10.3389/fpubh.2024.1295128

**Published:** 2024-05-02

**Authors:** Chava Pollak, Joe Verghese, Helena M. Blumen

**Affiliations:** ^1^Department of Medicine, Albert Einstein College of Medicine, Bronx, NY, United States; ^2^Department of Neurology, Albert Einstein College of Medicine, Bronx, NY, United States

**Keywords:** loneliness, physical activity, active aging, psychosocial, aging, function

## Abstract

**Background:**

Physical activity is associated with improved health and function in older adults, yet most older adults are sedentary. Loneliness is associated with decreased physical activity at the cross-section, but longitudinal studies are scarce. We examined longitudinal associations between loneliness and physical activity—and whether they were modified by marital status and network size (the number of children, relatives, and friends a person interacts with at least once a month).

**Methods:**

We analyzed data from 1,931 older adults without dementia at baseline from the Rush Memory and Aging Project with a mean follow-up of 4.8 years (mean age 79.6 ± 7.7, 74.9% women). Loneliness was assessed using the de Jong Gierveld Loneliness Scale. Physical activity was assessed as the frequency with which participants engaged in five categories of activities (e.g., walking, gardening, calisthenics, bicycling, and swimming). Linear mixed effects models examined associations between baseline loneliness and change in physical activity over time after adjusting for demographics, depressive symptoms, global cognition, disability, network size, marital status, social support, and social and cognitive activities. We assessed for effect modification by marital status and network size.

**Results:**

Associations between loneliness and physical activity differed by marital status. In widowed individuals, baseline loneliness was associated with a 0.06 h/week greater decrease in physical activity per year compared to those who were not lonely (*p* = 0.005, CI -0.1, 0.02)—which equaled a 150% decrease in physical activity per year. Loneliness did not predict a statistically significant decrease in physical activity in married or unmarried individuals.

**Discussion:**

Loneliness is associated with decreased physical activity in widowed older adults and should be considered in the design of interventions to prevent or slow the decline in physical activity and promote healthy aging.

## Introduction

Loneliness is a subjective, negative feeling of being alone and is associated with cognitive and functional decline and an increased risk of Alzheimer-type dementia and mortality (1–5). Loneliness is a public health problem in the United States, with an estimated prevalence of 43.2% in middle-aged and older adults (6). Loneliness is associated with decreased physical activity in older adults cross-sectionally; however, the underlying mechanisms for this association are unclear (7, 8). Additionally, longitudinal studies are scarce and conducted in small samples with short follow-up periods (7–9). Furthermore, associations between physical activity and loneliness are often studied without considering whether structural measures of social connection, such as social network size and marital status, influence such associations (10).

Increased physical activity is associated with several positive health outcomes in older adults, including improved cognitive function (11), reduced fall risk (12), and reduced risk of disability (13) and mortality (14). However, nearly 30% of middle-aged and older adults are sedentary, and physical inactivity increases with age (15). The Centers for Disease Control and Prevention (CDC) recommends a minimum of 150 min or 2.5 h per week of moderate-intensity physical activity (16). According to data from US national surveys, only between 27.3 and 44.3% of older adults met recommended physical activity levels; men were more active than women, racially minoritized groups were less active compared to Caucasians, and activity declined with age (17). Physical inactivity and sedentariness have increased since the onset of the COVID-19 pandemic (18), and sedentary time is associated with dose-related increased risk of chronic disease and all-cause mortality (19). Changes in physical activity patterns and sedentary behavior due to the COVID-19 pandemic demand attention to predictors of decreased physical activity as potential targets for intervention.

Lonely individuals are more likely to engage in negative health behaviors such as decreased physical activity (20). The Social Control Theory explains the association between loneliness and health behaviors through the influence of social cues on behavior choices (21). Individuals with large social networks or who live with a spouse or partner may theoretically have people around them who directly regulate their health behaviors or indirectly regulate behaviors through role modeling or reinforcing acceptable health-behavior norms (21). The purpose of this study was to examine longitudinal associations of baseline loneliness and physical activity in a sample of community-dwelling older adults. We also examined whether network size and marital status modified such associations based on the theoretical premise of the social control theory.

## Methods

### Study participants

This study was a secondary analysis of data from the Rush Memory and Aging Project (MAP), a clinical-pathologic cohort that began enrolling participants in 1997 (22, 23). The only inclusion criteria for the cohort were annual health assessments and anatomical gift donation at death. At the time, the data were obtained, 2,252 participants were enrolled in the study, and over 98% of them were enrolled before 2020. Participants included lay people recruited primarily from retirement communities in Northeastern Illinois to enable the participation of frail older adults and maintain high rates of follow-up and autopsy (22). We excluded 120 participants with dementia at baseline as their experience of loneliness and physical activity is likely different than individuals without dementia, and the reliability of responses on self-report items such as loneliness or physical activity may differ in individuals with dementia. An additional 201 participants were excluded for missing baseline loneliness measures. This left 1,931 participants for this analysis with a mean follow-up time of 4.8 years (SD 4.2). The MAP study was approved by the Rush Medical Center Institutional Review Board. Ethical approval for secondary analyses was obtained from the Albert Einstein College of Medicine Institutional Review Board.

### Physical activity

Physical activity was assessed using questions adapted from the 1985 National Health Interview Survey (24). Participants were asked whether they engaged in five categories of activities within the past 2 weeks and the average number of minutes that they spent doing each reported activity: (1) walking for exercise, (2) gardening or yard work, (3) calisthenics or general exercise, (4) bicycle riding, and (5) swimming or water exercise. Minutes in each activity were summed up and expressed in hours of activity per week.

### Loneliness

Loneliness was assessed using a modified, 5-item de Jong Gierveld Loneliness Scale. The de Jong Gierveld Loneliness Scale is a valid and reliable tool that assesses social and emotional loneliness (25). In this study, loneliness was characterized by the following: (1) I experience a general sense of emptiness, (2) I miss having people around, (3) I feel like I do not have enough friends, (4) I often feel abandoned, and (5) I miss having a really good friend. Participants were asked to rate agreement with each item on a 5-point Likert scale. Individual item scores were averaged to obtain total scores ranging from 1 to 5. Higher values indicated higher levels of loneliness. Loneliness was considered a continuous variable in these analyses.

### Effect modifiers

Marital status and network size are structural measures of social connection and fit with the theoretical framework of this study, which states that structural measures of social connection may modify the relationship between loneliness—a functional measure of social connection—and health outcomes. Marital status was assessed at baseline with questions inquiring if the participant was ever married and, if so, their current marital status. Responses were registered as never married, married, widowed, divorced, or separated. For these analyses, the variable was grouped into three categories: married for participants who reported they were currently married, widowed for participants who reported they were currently widowed, and unmarried for participants in any other category. Network size was quantified as the total number of children, relatives, and friends that a participant interacted with at least once a month (26).

### Covariates

Covariates were selected based on prior associations with loneliness and/or physical activity. Age and gender are associated with loneliness, and the subjective experience of loneliness may differ by both factors (27, 28). Age in years was computed from self-reported date of birth and date of clinical evaluation. Gender was self-reported as male or female. Race and education may represent proxies for socioeconomic status and might influence the availability of leisure time to engage in physical activity and the lived environment with available space or facilities to engage in activities. Race was based on self-report and included 7 categories: white, Black/African American, American Indian or Alaskan Native, Native Hawaiian or Other Pacific Islander, Asian, Other, or Unknown. Since over 90% of the sample self-identified as white, we collapsed the race variable into 3 categories: white, Black/African American, and other. Years of education were based on the number of self-reported years of schooling. Socioeconomic status is also an important risk factor for loneliness (29). Loneliness is associated with depressive symptoms and disability (29, 30). Additionally, comorbidities and physical disability may influence both loneliness and physical activity (31–34) as the individual may be preoccupied with health problems, limited by functional abilities, and require more tangible support from social relationships. Disability was assessed with the 3-item Rosow-Breslau scale that measures the ability to do 3 activities: heavy work around the house, walking up and down stairs, and walking half a mile without help (35). Responses were registered as 0–no help, 1–help, and 1–unable to do. Responses were summed with higher scores indicating a higher level of disability. Depression was assessed with a modified 10-item Center for Epidemiologic Studies Depression Scale (CES-D) (36). An overall depression score was computed as the sum of symptoms experienced, with higher scores indicating higher levels of depressive symptoms. For the analyses, the depression score was re-calculated, excluding the loneliness item, so as not to over-adjust for loneliness. Comorbidities included a composite measure of the sum of 7 medical conditions: hypertension, diabetes, heart disease, cancer, thyroid, head injury, and stroke. Frequency of social activities was assessed using a previously established 6-item composite measure of social activity (37): (1) go to restaurants/sporting events, (2) day trips/overnight trips, (3) volunteer work, (4) visit friends or relatives, (5) participate in groups (e.g., senior center), and (6) attend religious services. Participants were asked how often they engaged in these activities over the past year. Items were rated on a 5-point scale, with higher values indicating more frequent participation. Items were summed and averaged to yield a composite social activity score. This measure was previously associated with motor decline in older adults (38). Cognitive activity was assessed by frequency of participation in 7 cognitively stimulating activities during the past year using a structured questionnaire and rated on a 5-point scale: (1) reading, (2) visiting the library, (3) reading newspapers, (4) reading magazines, (5) reading books, (6) writing letters, and (7) playing board games/puzzles. Items were summed and averaged to yield a composite score of cognitive activity frequency, with higher scores indicating more frequency participation. This measure was shown to have adequate internal consistency in prior studies and was associated with higher levels of education and cognitive function (39, 40).

## Data analysis

**Figure 1:**
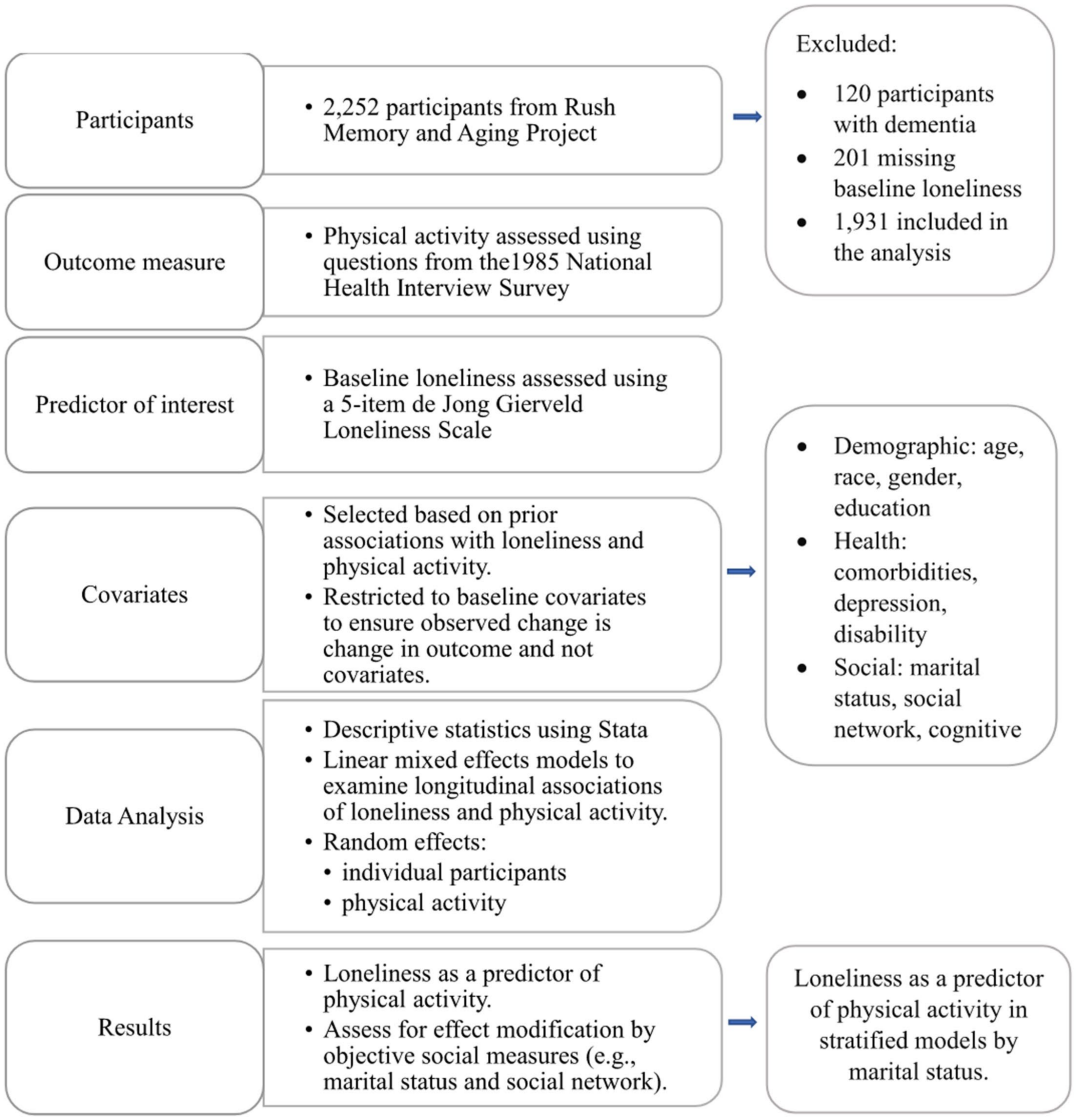
Summary of study procedure and analysis approach.

A summary of the study procedure and analysis approach is presented in Figure 1. We ran descriptive statistics for the sample overall and by unmarried, married, and widowed status. We used one-way ANOVA and Kruskal–Wallis tests for comparisons of continuous variables and chi-square tests for comparisons of categorical variables to describe the sample. We included all participants with baseline loneliness measures in the analysis, regardless of follow-up time, to maximize the available data. As many of our covariates are correlated, we ran a simple regression model to test for collinearity. We applied linear mixed-effects models to examine the longitudinal associations between loneliness and physical activity. We included the follow-up year as our time variable. We modeled physical activity as the outcome and baseline loneliness as the predictor. We restricted our analyses to baseline loneliness to examine the rate of change in physical activity over time and not change in loneliness. We included an interaction term for loneliness and time to model the rate of change in physical activity predicted by baseline loneliness. We assessed the linearity of physical activity trajectories over time using likelihood ratio tests to compare linear and quadratic models. Covariates were chosen for inclusion based on prior associations with loneliness and physical activity and biological relevance, including demographic covariates (e.g., age, race, gender, and education), health variables (e.g., comorbidities, depression, and disability), and social and cognitive measures (e.g., social and cognitive activity and social support). We used baseline covariate data to be concurrent with loneliness and to ensure that any observed change in physical activity reflected a change in physical activity and not a change in covariates. Variables that did not significantly contribute to the model but were biologically important and/or relevant to the theoretical framework of the study remained in the final model. We then added random effects to the model based on the Akaike information criterion (AIC) to choose the best model. Individual participants were included as random effects to account for non-independence in measures within participants. We modeled physical activity as a random effect to allow for physical activity trajectories to vary by individual participants. We modeled an autoregressive covariance structure to reflect a decreasing correlation between measures over time, as expected in longitudinal studies. We assessed for effect modification between loneliness—a functional social connection measure—and structural social connection measures (e.g., network size and marital status) using product terms for loneliness and each covariate. Since we found effect modification by marital status, we present stratified models for effects in unmarried, married, and widowed individuals separately. All statistical tests were two-tailed, and an *a priori p* < 0.05 was considered statistically significant. Data were inspected graphically and statistically, and model assumptions were found to be adequately met. Stata (StataCorp LLC, College Station, TX) version 17.0 was used for all analyses.

## Results

### Baseline characteristics of participants

Descriptive statistics for participants overall and by unmarried and married are displayed in Table 1. Approximately 20% of participants were unmarried at baseline, 39.6% were married, and 41.5% were widowed; 69.2% of men (*n* = 334) and 30% of women (*n* = 431) were married at baseline, and 20% of men and 48.6% of women were widowed at baseline. The mean age of participants was 79.6 ± 7.7. Participants were 74.9% female and 92.8% Caucasian, with an average of 15 years of education (SD 3.4). Participants had a median of 5 contacts in their social network (Interquartile range (IQR) 3, 9). Participants were generally well-supported and active, with a mean of 3.5 h of physical activity per week (SD 3.8). They were also cognitively and functionally intact and had low comorbidities and depressive symptoms. Compared to married and unmarried individuals, widowed participants were significantly older and had significantly lower cognitive function. There were statistically significant differences in ethnicity, education, cognition, physical function, and depression by marital status. Additionally, social factors, including network size, social support, physical activity, cognitive activity, and loneliness, also significantly differed between married, unmarried, and widowed participants.

**Table 1:** Baseline characteristics of participants overall and by married and unmarried.

	Overall (*n* = 1,931)	Unmarried (*n* = 365)	Married (*n* = 765)	Widowed (*n* = 800)	*p*-value
Age (years)	79.6 ± 7.7	76.1 ± 8.4	77.9 ± 7.2	82.9 ± 6.5	<0.001
Female, % (*n*)	74.9 (1, 447)	85.8 (313)	56.3 (431)	87.9 (703)	<0.001
Ethnicity, % (*n*)					<0.001
Caucasian	92.8 (1, 792)	84.9 (310)	95.7 (732)	93.8 (750)	
African American	5.7 (110)	11.5 (42)	3.7 (28)	4.9 (39)	
Other	1.5 (29)	3.6 (13)	0.7 (5)	1.4 (11)	
Education (years)	15.0 ± 3.4	15.1 ± 3.6	15.6 ± 3.4	14.4 ± 3.0	<0.001
Structural Social Factors					
Social Network Size, median (IQR)	5 (3, 9)	5 (2, 8)	6 (3, 10)	5 (3, 9)	<0.001
Functional Social Factors					
Social Support, median (IQR), range 1–4	4.4 (4, 5)	4 (4, 4.8)	4.8 (4, 5)	4.3 (4, 5)	<0.001
Physical Activity (hours/week)	3.5 ± 3.8	3.4 ± 3.7	3.7 ± 4.0	3.4 ± 3.6	0.01
Social Activity, range 1–6	2.6 ± 0.6	2.6 ± 0.6	2.7 ± 0.6	2.7 ± 0.6	0.24
Cognitive Activity, range 1–7	3.2 ± 0.7	3.1 ± 0.7	3.2 ± 0.6	3.1 ± 0.7	<0.001
Quality Social Factors					
Loneliness, range 1–5	2.2 ± 0.6	2.3 ± 0.6	2.1 ± 0.6	2.3 ± 0.6	<0.001
Cognitive Function					
Global cognitive function summary	0.08 ± 0.6	0.1 ± 0.6	0.2 ± 0.5	−0.04 ± 0.5	0.03
Physical Function, median (IQR)					
Disability	0 (0, 1)	0 (0, 1)	0 (0, 1)	1 (0, 1)	<0.001
Psychological Factors, median (IQR)					
Depressive symptoms (CES-D), range 1–10	0 (0, 2)	1 (0, 2)	0 (0, 1)	1 (0, 2)	<0.001
Comorbidities					
Self-reported conditions	1.4 ± 1.1	1.5 ± 1.0	1.3 ± 1.0	1.5 ± 1.1	0.53

Values expressed in means, SD unless otherwise noted. SD, standard deviation; IQR, interquartile range; Disability was measured by the Rosow-Breslau scale; Comorbidities were assessed by a composite score of 7 self-reported conditions: hypertension, diabetes, heart disease, cancer, thyroid disease, head injury, and stroke.

### Baseline loneliness and physical activity by marital status

**Figure 2:**
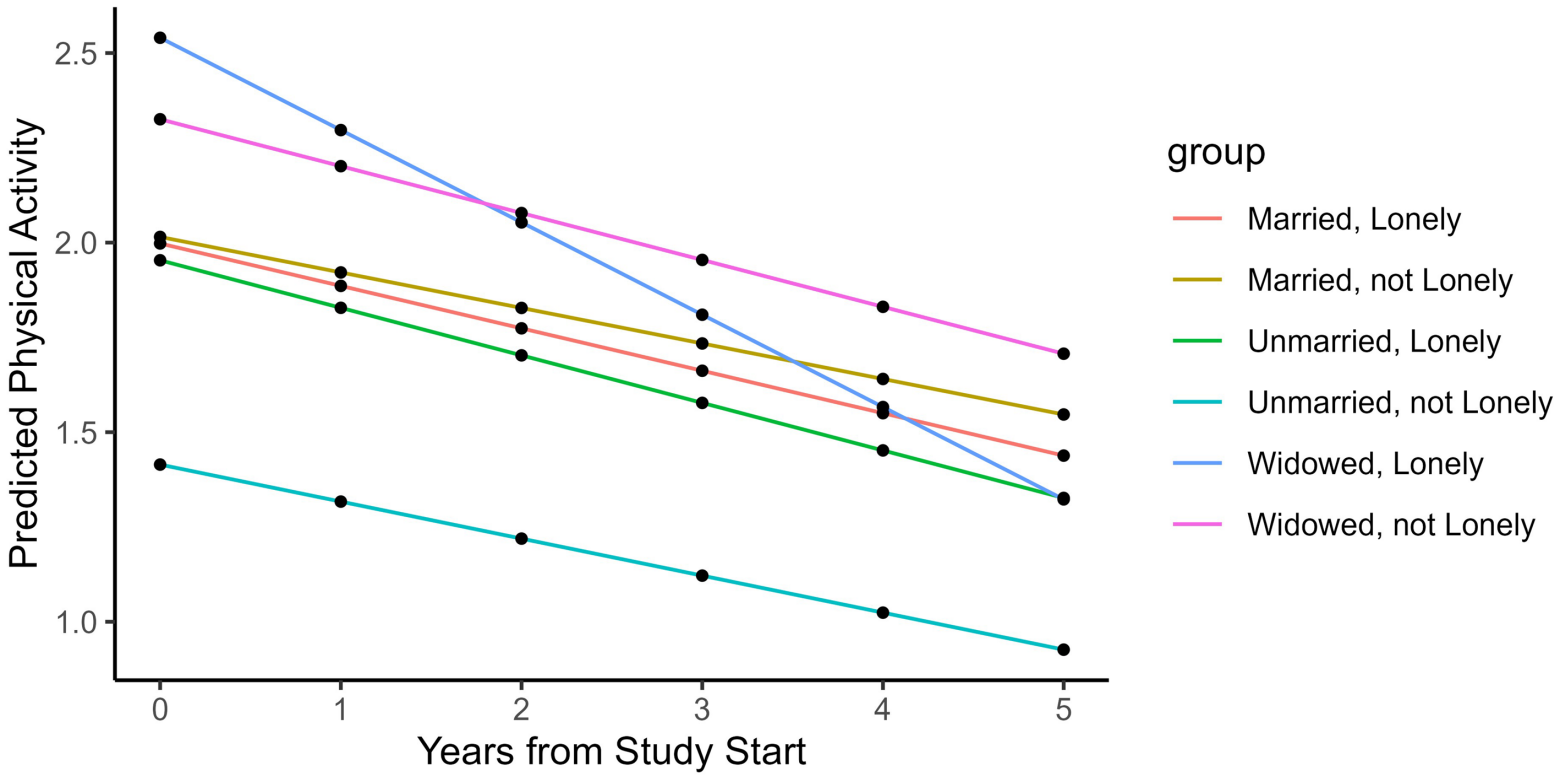
Model prediction for physical activity over time. "Assuming an 80-year old woman with all other covariates as 0 and a loneliness score of 4 if she is lonely and 2 if she is not lonely.

The effects of loneliness on physical activity over time differed between unmarried, married, and widowed participants (Table 2). Baseline loneliness predicted significant declines in physical activity in widowed but not married or unmarried participants. In widowed participants (*n* = 800), baseline loneliness predicted a 0.06 h—or 3.6 min—per week decrease in physical activity per year (*p* = 0.1, CI -0.11, 0.01). The 3.6-min weekly decrease in physical activity per 1-point increase in loneliness equaled a 150% decrease in physical activity per year. Widowed participants had a non-significant decrease of 0.004 h per week in physical activity per year (*p* = 0.95, CI -0.11, 0.11); however, loneliness predicted a significant decline in physical activity in widowed individuals. Physical activity decreased by 0.07 h/week and 0.009 h/week in the unmarried and married groups, respectively; however, this decrease was not statistically significant (*p* = 0.34, CI -0.21, 0.07 and p = 0.3, CI -0.22, 0.07, respectively). Among married (*n* = 765) and unmarried (*n* = 365) individuals, loneliness did not predict a decline in physical activity over time over and above the non-significant yearly decline (*p* = 0.79, CI -0.08, 0.06, *p* = 0.65, CI -0.7, 0.05, respectively). These results are graphically depicted in Figure 2 over 5 years of follow-up, as most participants were followed for less than 5 years. As demonstrated in the figure, physical activity declined over time in all groups. Loneliness influenced physical activity trajectories in all groups; however, widowed and lonely individuals had the steepest decline in physical activity over 5 years. Loneliness was not significantly associated with physical activity cross-sectionally in any group. The effects of loneliness on physical activity did not differ by gender or network size in stratified or unstratified models.

**Table 2:** Loneliness is associated with rate of change in physical activity in widowed but not married or unmarried older adults.

	Unmarried (*n* = 365)	Married (*n* = 765)	Widowed (*n* = 800)
Model term	*b*(95% CI)	*b*(95% CI)	*b*(95% CI)
	*p-*value	*p-*value	*p-*value
Loneliness x time*	−0.01 (−0.07, 0.05)	−0.009 (−0.08, 0.06)	−0.06 (−0.11, 0.01)
	*0.65*	*0.79*	0.01**
Time	−0.07 (−0.2, 0.07)	−0.08 (−0.22, 0.07)	0.004 (−0.11, 0.11)
	*0.34*	*0.3*	*0.95*
Loneliness	0.27 (−0.3, 0.9)	−0.009 (−0.52, 0.5)	0.11 (−0.28, 0.5)
	*0.38*	*0.97*	*0.59*
Age	−0.06 (−0.1, −0.02)	−0.08 (−0.11, −0.04)	−0.03 (−0.11, −0.01)
	0.002**	<0.001**	0.01**
Gender	−0.82 (−1.8, 0.1)	−0.59 (−1.05, −0.13)	−0.25 (−0.76, 0.27)
	*0.09*	<0.01**	*0.35*
Race	0.03 (−0.7, 0.8)	−0.25 (−1.07, 0.56)	0.11 (−0.44, 0.65)
	*0.93*	*0.54*	*0.71*
Education	0.09 (−0.004, 0.19)	0.07 (−0.001, 0.14)	0.03 (−0.03, 0.09)
	*0.06*	*0.06*	*0.31*
Comorbidities	−0.49 (−0.8, −0.2)	−0.17 (−0.37, 0.04)	−0.01 (−0.17, 0.14)
	0.001**	*0.11*	*0.89*
Disability	−0.37 (−0.7, −0.04)	−0.72 (−0.99, −0.45)	−0.41 (−0.59, −0.24)
	0.03**	<0.001**	<0.001**
Depressive symptoms	−0.14 (−0.4, 0.06)	−0.17 (−0.35, 0.01)	−0.15 (−0.27, −0.03)
	*0.17*	*0.07*	0.02**
Social Network	0.03 (−0.04, 0.1)	0.02 (−0.009, 0.06)	0.009 (−0.02, 0.04)
	*0.44*	*0.16*	*0.55*
Social Support	−0.26 (−0.7, 0.2)	−0.06 (−0.43, 0.31)	−0.11 (−0.3, 0.2)
	*0.26*	*0.77*	*0.44*
Social Activity	0.43 (−0.1, 1.0)	0.21 (−0.21, 0.63)	0.54 (0.37, 0.16)
	*0.15*	*0.33*	0.001**
Cognitive Activity	0.65 (0.17, 1.1)	0.31 (−0.11, 0.72)	0.02 (−0.23, 0.27)
	0.008**	*0.15*	*0.89*

CI, confidence interval. *Represents annual rate of change in physical activity per one unit increase in loneliness. Other terms represent cross-sectional associations of variables with physical activity. **Represents statistically significant result at the a priori cutoff of *p* = 0.05. The bolded values are to draw attention to significant values. The *p* values are in italics to differentiate them from other values.

## Discussion

The main finding of this study was that in a cohort of 1,931 individuals without dementia at baseline, loneliness predicted decreased physical activity in widowed but not unmarried or married participants. This finding aligns with the theoretical basis for this study that a structural measure of social connection modifies the association between loneliness—a functional measure of social connection—and physical activity. These results highlight widowed older adults as a high-risk group that should be considered in the assessment and risk stratification of older adults and in the design of interventions for loneliness and physical activity.

Our findings are consistent with previous studies that showed loneliness predicted decreased physical activity cross-sectionally and longitudinally (7–9, 20). Loneliness was also associated with an increased likelihood of discontinuation of physical activity over time (7). In a longitudinal study of 3,392 participants with 77% of participants between the ages of 52 and 69 years, loneliness measured with the UCLA Loneliness Scale was associated with decreased self-reported physical activity over 10 years of follow-up (20). Our results extend the literature on associations of loneliness and physical activity in an older sample of community-dwelling older adults with a mean age of 79.6 years and up to 20 years of follow-up. While some cross-sectional studies assessed associations of loneliness and physical activity using objective physical activity measures (8), most used self-reported physical activity measures (7–9, 20, 41). Self-reported physical activity might be overestimated due to social acceptability bias or recall bias; thus, our results might be underestimated. Studies on associations between loneliness and objectively measured physical activity reflect a gap in the literature that requires further research.

We found that marital status modified associations between loneliness and physical activity. The inclusion of structural measures of social connection in our analysis responds to a gap in understanding how loneliness is influenced by structural measures (5). Importantly, while structural measures of social connection may be correlated with loneliness, they are distinct constructs. Loneliness reflects an individual’s satisfaction with the quality or quantity of social relationships. In other words, an individual who is alone (e.g., lives alone or has a small network size) may not feel lonely, while some who are surrounded by others (e.g., married) may feel very alone (5). Our findings highlight that widowed individuals are at a high risk of loneliness and physical inactivity and, thus, are an important group to target for interventions. We frame our understanding of this finding in the Social Control Theory of behavior, where individuals are influenced by those around them in terms of health choices, which points to a potential mechanism for how loneliness interventions can be impactful on physical activity in older adults (21). We did not find effect modification by network size, which suggests that simply increasing the objective number of social contacts might not be adequate to address the effects of loneliness on physical activity because individuals may be surrounded by others and still feel lonely (5). This is an area in which assessment of relationship quality might provide clarification because not all relationships are positive, and negative relationships may negatively impact health behaviors and health outcomes (42).

We found that loneliness predicted decreased physical activity in widowed participants but not in participants who were married or unmarried. Widowhood is a great source of trauma and has significant health and mortality effects, particularly in the early bereavement period (43, 44). Data from 34,777 individuals from the Health and Retirement Study (HRS) aged 51 years and older showed widowhood increased mortality risk, and the risk differed by race and gender (45). Our findings add to the literature on widowhood and health outcomes and suggest a possible mechanism for the relationship related to effects on health behaviors such as physical activity. Our understanding of these effects is limited as our data did not include repeated measures of marital status or information regarding transitions in marital status during the study. Systematic reviews on the influence of life events and transitions on physical activity across the lifespan suggest that physical activity levels increase immediately post-widowhood; however, these trajectories may not be sustained, and effects may differ between men and women (46–48). Our findings show, however, that in lonely, widowed individuals, physical activity significantly declined over time after adjusting for possible confounders such as disability, comorbidities, or depression, highlighting the negative health effects of loneliness.

Our findings are also limited by the lack of a marital quality measure. Marital quality influences feelings of loneliness (49) and might affect associations of loneliness and physical activity in married or widowed individuals, either positively or negatively (42, 50). In a study by Hsu et al. spouses of individuals living with dementia with good marital quality had more loneliness as a result of their spouse’s change in cognition compared to those with low marital quality (49). Furthermore, a sub-sample from the HRS with a mean age of 73 (SD 8.7) and better marital quality who were recently widowed showed increased distress and depressive symptoms compared to people with poor marital quality (51). It is possible marital quality might similarly affect loneliness and physical activity. However, our findings cannot extrapolate on this potential due to limitations of the data. The effects of marital quality on associations of loneliness and physical activity require further research. Additionally, we lacked data regarding living arrangements and cohabitation, which may have similar effects on behavior as marriage (21). Thus, our results bear repeating in other samples with consideration for living arrangements. Note also that our results do not necessarily apply to other validated and recommended measures of network size and social activity (5)—although some questions overlap. Our social network measure, for example, is similar to the social network index (52), which quantifies the number of people a person interacts with biweekly across 12 different types of social relationships (spouse, parent, child, child-in-law, close relative, close friend, religious group member, student, employee, neighbor, volunteer, and group member).

We report findings on physical activity based on self-report, which is inherently error-prone due to recall bias. Self-reported physical activity tends to be overestimated and sedentary time underestimated, which can influence estimates of effects (53, 54). Self-reported physical activity also does not account for activity accumulated through everyday tasks that are not routinely identified as formal exercise. The physical activity measure used in this study, while it is self-reported, inquired about the frequency of five activity categories, which includes more detail than a question regarding overall activity or overall intensity of activity; however, not all activities individuals might engage in were included. Due to the nature of cohort data, temporality cannot be established, and we cannot rule out reverse causality. It is possible that decreased physical activity, whether it is related to cognitive or physical function or other reasons, leads to decreased social interactions and, therefore, increased loneliness. The current analyses were restricted to baseline loneliness and rate of change in physical activity over time to examine trajectories of physical activity predicted by loneliness. The relationship between loneliness and physical activity, however, may be bidirectional, and examination of whether baseline physical activity predicts change in loneliness over time is an area of future study. Additionally, the characteristics of our sample limit the generalizability of our findings. Notably, our findings on loneliness and physical activity in older adults are relevant to a cohort of much older (mean age 79.6 years), relatively healthy, mostly Caucasian, mostly female, and highly educated cohort of US older adults. Additionally, on average, our cohort exceeded the recommended 2.5 h of weekly physical activity by an hour at baseline. It is possible that, since participants had higher activity levels at baseline, they had steeper age-related declines in physical activity over time compared to older adults who may already be less active. This is a common problem with cohort studies, particularly volunteer cohorts. It is imperative that future research in this area is inclusive of the populations that we serve and are particularly affected by these issues.

Our study has many strengths that lend confidence to our findings. We included a large, well-characterized sample of older adults with many years of follow-up. The study uniquely enjoys high follow-up participation, which reduces attrition-related bias. We also included a wide range of covariates that might influence our results, and we showed that associations of loneliness and physical activity persisted after adjusting for these potential confounders. Finally, our study is grounded in a theoretical framework to support our hypotheses. These findings extend the literature on loneliness and physical activity and highlight many avenues for future research to further our understanding of these associations.

## Implications

Our results highlight loneliness as a health imperative associated with decreased physical activity over time in widowed, community-dwelling older adults. Decreased physical activity is associated with a range of negative health outcomes in older adults, including, but not limited to, disability and functional decline. Maintenance of function is crucial for independent living in older adults. These results support loneliness as an intervention target to prevent a decline in physical activity and physical function in community-dwelling older adults, particularly those who are objectively alone.

Physical activity interventions conducted in a group setting and other active interventions with social components have the potential to target both loneliness and physical activity. Some physical activity programs, such as SilverSneakers, are covered by health plans and can be accessed by Medicare Advantage beneficiaries (55) and others are offered for nominal fees via older adult centers available to local older adults in a group setting. Additionally, the use of virtual physical activity programs has proliferated since the onset of the pandemic and has the potential to connect older adults who are less mobile or live in remote settings (56). Availability and access to these types of programs vary, however. Given that addressing loneliness and physical activity are crucial for healthy aging, it is a health policy imperative to improve the accessibility and reach of these programs.

## Conclusion

In a sample of 1,931 older adults without dementia at baseline, loneliness predicted a decline in physical activity in widowed but not married or unmarried individuals. These results underscore the importance of psychosocial factors and physical activity in aging and suggest the need for interventions and policy investment in the prevention and treatment of loneliness to promote healthy aging.

## Data availability statement

The data analyzed in this study is subject to the following licenses/restrictions: the data was shared through a data sharing agreement and is not owned by any of the authors. Requests to access these datasets should be directed to Gregory_Klein@rush.edu.

## Ethics statement

The studies involving humans were approved by Rush University Medical Center and Albert Einstein College of Medicine. The studies were conducted in accordance with the local legislation and institutional requirements. The participants provided their written informed consent to participate in this study.

## Author contributions

CP: Conceptualization, Data curation, Formal analysis, Funding acquisition, Investigation, Methodology, Writing – original draft, Writing – review & editing. JV: Conceptualization, Funding acquisition, Methodology, Project administration, Resources, Supervision, Validation, Writing – review & editing. HB: Conceptualization, Funding acquisition, Investigation, Methodology, Project administration, Resources, Supervision, Validation, Writing – review & editing.
