# Supplementary figures and images for: Loneliness predicts decreased physical activity in widowed but not married or unmarried individuals
Source: Front Public Health. 2024 May 2;12:1295128. doi: 10.3389/fpubh.2024.1295128 (PMC11096491; doi:10.3389/fpubh.2024.1295128)

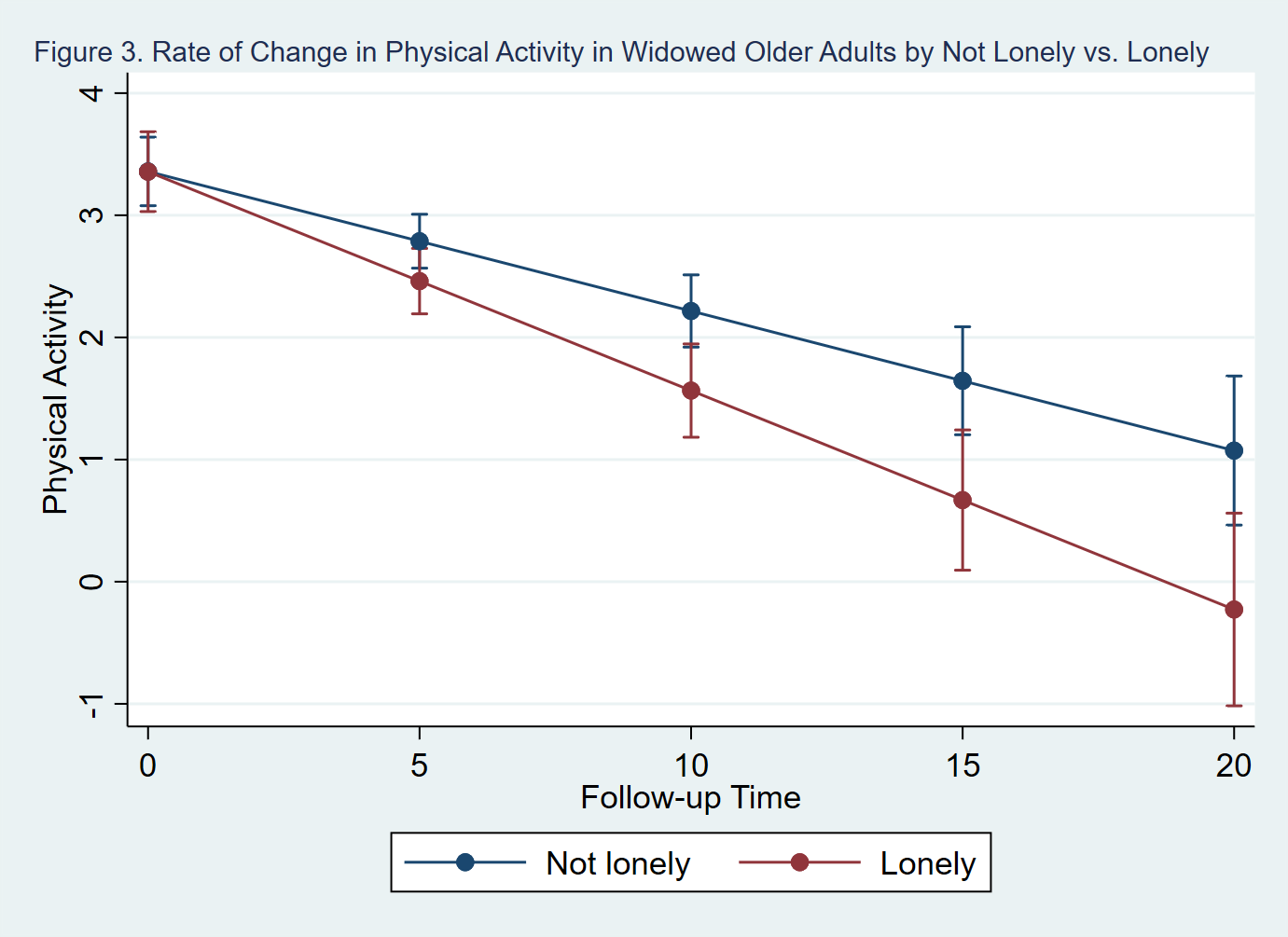

Supplement: Supplementary file 1 [file Image_1.TIF]
